# Increased breast cancer risk in women with neurofibromatosis type 1: a meta-analysis and systematic review of the literature

**DOI:** 10.1186/s13053-019-0110-z

**Published:** 2019-03-25

**Authors:** Lorena P. Suarez-Kelly, Lianbo Yu, David Kline, Eric B. Schneider, Doreen M. Agnese, William E. Carson

**Affiliations:** 1 Division of Surgical Oncology, Department of Surgery, The Ohio State University Wexner Medical Center, Columbus, OH 43210 USA; 2 Department of Biomedical Informatics, The Ohio State University, Columbus, OH 43210 USA; 3 Division of Critical Care, Trauma & Burn, Department of Surgery, The Ohio State University, Columbus, OH 43210 USA; 4 The Arthur G. James Comprehensive Cancer Center and Richard J. Solove Research Institute, The Ohio State University, N924 Doan Hall 410 W. 10th Ave, Columbus, OH 43210-1228 USA

**Keywords:** Breast cancer, Neurofibromatosis type 1, NF1

## Abstract

**Background:**

Neurofibromatosis type 1 (NF1) is a cancer predisposing syndrome. Studies suggest that women < 50 years old (y.o.) with NF1 have an increased breast cancer (BC) incidence and BC associated mortality. However, this has not been widely recognized secondary to small study populations.

**Methods:**

A systematic literature review was conducted through database searches for BC and NF1: 3456 articles identified, 166 reviewed, 58 used for descriptive analysis and 4 utilized for meta-analysis. Fisher’s exact tests, Kaplan-Meier curves and random-effects meta-analysis models were used for analysis.

**Results:**

Two hundred eighty-six cases of NF1 and female BC were identified with a median age of 46 years at diagnosis; 53% were <  50. Peak age of BC diagnosis was between 34 to 44 years. Women < 50 y.o. presented with more advanced disease vs. those ≥50 (56% vs. 22% stage III-IV, respectively; *p* = 0.005). Median survival for the entire cohort was 5 years vs. the reported median BC survival of over 20 years in the general population using the SEER database. Median age at BC death was 48.5 years; 64% of deceased patients were <  50. Meta-analysis of a total of 4178 women with NF1 revealed a BC standardized incidence ratio (SIR) of 3.07 (95%CI 2.16–4.38) for women with NF1 vs. the general population. Women < 50 y.o. demonstrated a higher SIR of 5.08 (95%CI 3.77–6.81) compared to 1.92 (95%CI 1.40–2.63) if ≥50 y.o.

**Conclusions:**

This systematic literature review and meta-analysis suggests that women with NF1 <  50 y.o. have a five-fold increased risk of BC, present with more advanced disease, and may have an increased BC related mortality. Increased awareness and implementation of recent National Comprehensive Cancer Network early BC screening guidelines for this high-risk patient population is essential. Additional evaluation on the influence of *NF1* gene mutations identified in patients undergoing hereditary cancer genetic testing on breast cancer risk in individuals without clinical evidence of NF1 is needed.

**Electronic supplementary material:**

The online version of this article (10.1186/s13053-019-0110-z) contains supplementary material, which is available to authorized users.

## Background

Neurofibromatosis type I (NF1) is one of the most common autosomal dominant genetic disorders in humans and has a reported incidence of 1 in 2000 to 3000 live births with most population based studies demonstrating a prevalence of the clinical diagnosis in 1 in 3000 to 1 in 5000 individuals [1–5]. Clinical diagnosis of NF1 is made following the National Institutes of Health guidelines in which the patient should have two or more of the seven cardinal diagnostic criteria: six or more café-au-lait spots (> 5 mm pre-puberty, > 15 mm post-puberty); two or more neurofibromas of any type, or one or more plexiform neurofibroma; freckling in the axillae or groin; optic glioma; two or more Lisch nodules; dysplasia of the sphenoid, or dysplasia or thinning of long bone cortex; or first degree relative with NF1 [6, 7]. NF1 is caused by a mutation in the neurofibromin 1 (*NF1*) gene located on chromosome 17q11.2, with a heterozygous mutation having nearly 100% penetrance [6]. The *NF1* gene is 282.7 kilobase pairs in length, contains 60 exons, and encodes neurofibromin [7, 8]. Given its large size, the *NF1* gene has one of the highest rates of spontaneous mutations in the entire human genome [6].

Neurofibromin functions as a tumor suppressor gene through regulation of Ras guanosine triphosphatase activity, inhibiting GTPase activation, and regulating cell proliferation and differentiation [6, 7]. Loss of neurofibromin results in uncontrolled cell proliferation and predisposition to the development of several cancers [8, 9]. The most commonly reported malignancies in patients with NF1 are gliomas and malignant peripheral nerve sheath tumors, however gastrointestinal stromal tumors, pheochromocytomas and childhood rhabdomyosarcoma have also been associated with NF1 [10–14]. Interestingly, somatic mutations of the NF1 gene are reported in 27.7% of all breast carcinomas and have been implicated as potential genomic drivers in the development of breast cancer [15, 16].

A link between NF1 and breast cancer has been suggested in several cohort and epidemiological studies [9, 17–22] and numerous cases of patients with NF1 presenting with breast cancer have been reported. The strength of the association between NF1 and the increased breast cancer risk remains uncertain due to the small study populations and differences in participants and methodological methods used in the previous studies. The objective of this study is to highlight the association between women with NF1 and an increased risk of breast cancer and to reinforce the importance for physician and patient education on the need for early breast cancer screening for this patient population. In the current study, we conducted a systematic review of the literature and meta-analysis of epidemiological studies to quantitatively assess the association between NF1 and the risk of breast cancer.

## Methods

### Literature sources and search strategy

The guidelines published by the Meta-analysis of Observational Studies in Epidemiology (MOOSE) group were followed to complete the meta-analysis (Additional file 1) [23]. A literature search was conducted using PubMed and PMC for all relevant studies published in English-language journals up to December 2015. We used the following MeSH and free-text terms in the search strategy: “Neurofibromatoses”, “Neurofibromatosis 1”, “genes, Neurofibromatosis 1”, and “Neurofibromatosis type 1” in combination with “breast neoplasms”, “breast cancer”, “malignancy”, “neoplasm”, “tumor”, or “cancer.” The search was restricted to studies in human beings and publications in English language. The reference lists of identified articles and relevant reviews were also checked for potentially eligible studies.

### Study selection

The studies were reviewed and case reports of patients with NF1 and breast cancer and observational studies that investigated the relationship between NF1 and breast cancer risk were reviewed. All studies, including case reports, were included in our descriptive analysis. Studies that met the following criteria were included in the meta-analysis: (i) case-control or cohort-based study design; (ii) investigation of the association between NF1 and breast cancer incidence; (iii) presented standardized incidence ratio (SIR), relative risk (RR), odds ratios (OR), or hazard ratio (HR) estimates with 95% confidence intervals (CI) or sufficient data with which to calculate these. The exclusion criteria were (i) abstracts without full texts, (ii) unpublished studies, (iii) lack of available data, (iv) male sex and (v) the following types of articles: news, previews, reviews, comments, and discussions.

### Data extraction and quality assessment

The following data from each included study were extracted: first author, publication year, country, study design, sample size, number of cases/controls, diagnostic criteria, age at time of breast cancer diagnosis, follow-up duration, breast cancer stage at time of diagnosis, breast cancer subtype, development of bilateral breast cancer, development of other primary cancers, development of metastatic breast cancer, survival outcome, and effect sizes (SIR, RR, OR, HR) with 95% CI and adjusted factors. Data were independently extracted and analyzed by two investigators and a final decision was reached by consensus. The methodological quality of the studies included in the meta-analysis was assessed using the Newcastle-Ottawa Scale (NOS) [24]. NOS scores of 0–3, 4–6, and 7–9 were regarded as low, moderate, and high quality, respectively [25].

### Comparison to population data

Rough comparisons of the NF1 patients with breast cancer identified in this study were made to the general population using data reported in the Surveillance Epidemiology and End Results (SEER) database for the years 1975–2012 [26]. Age distribution of incident cases and age distribution of deaths for female breast cancer reported in the SEER data base were compared to the age distribution of incident cases and age distribution of deaths of the female NF1 patients with breast cancer identified in this study using SEER data Table 1.11 and Table 1.13 [27, 28]. The relative survival of the female NF1 patients with breast cancer identified in this study was compared to the general population using SEER data Tables 4.14, 4.15, and 4.16 controlling for age and year of diagnosis for each patient [29–31].

### Statistical analysis

Descriptive statistics were provided to summarize patient characteristics and outcomes for the two NF1 age groups. For those variables in categories, Fisher’s exact test was used for testing across groups. Kaplan-Meier curves were used to estimate survival probabilities for the collected NF1 cases, and compared to the matched survival probabilities of general population from SEER database. To statistically combine estimates from studies that contained sufficient information, we used random effects meta-analysis models [32]. The log(SIR) and corresponding standard error from each study were combined to estimate the overall average SIR. The SIRs for the entire population, those under age 50 and those over age 50 were separately estimated. Each model was fit using the DerSimonian-Laird approach [33] as implemented in the MAd R package [34].

## Results

### Literature search and study selection

**Fig. 1:**
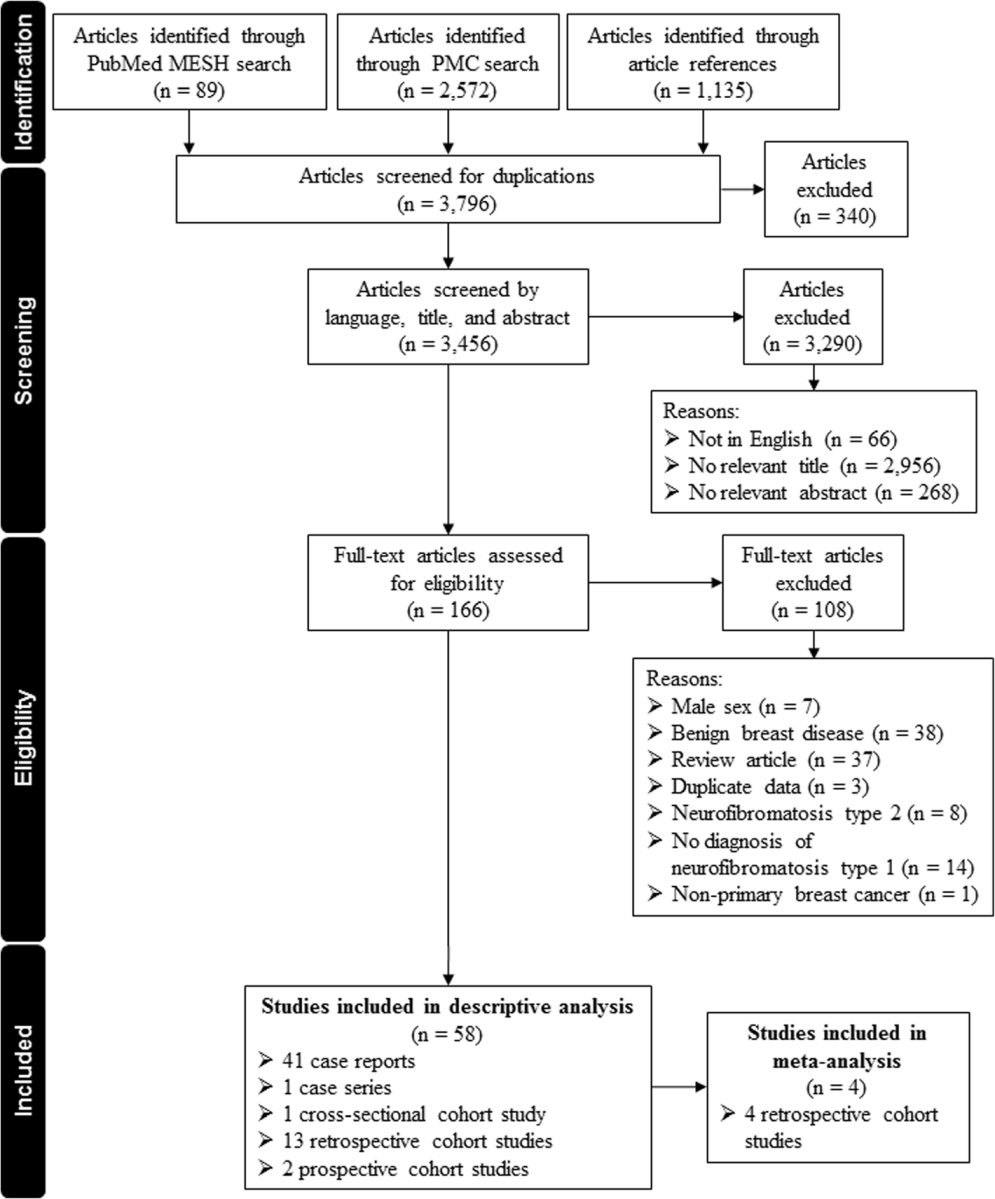
Flow-chart of study selection

The search strategy resulted in 3796 records: 89 from PubMed, 2572 from PMC and 1135 through examination of reference lists. After excluding duplicated reports, 166 full-text articles were identified on the basis of language, title, and abstract. After further evaluation, 101 records were excluded due to male sex, the lack of NF1 diagnosis, benign breast disease, review article, duplicate data, neurofibromatosis type 2, and non-primary breast cancer. A total of 58 eligible articles published between 1933 and 2015 were identified and were included in the descriptive analysis [9, 10, 17–22, 35–84]. Out of the 58 eligible articles, four meet the required inclusion criteria and were included in the meta-analysis [9, 18, 20, 21]. The study selection process is shown in Fig. 1.

### Descriptive analysis

Among the 58 studies included in the descriptive analysis, 41 were case reports, one was a case series, 13 were retrospective cohort studies, two were prospective cohort studies, and one was a cross-sectional cohort study. Two hundred and eighty-six cases of NF1 and female breast cancer were identified. The female NF1 patients with breast cancer originated from 23 different countries, with the majority of them originating from the United States of America (38%), United Kingdom (31%), and Japan (12%). Characteristics of these cases are described in Table 1.

**Table 1:** Breast Cancer Characteristics – all patients and by two age groups

	All Patients	< 50	≥ 50	*p*
N	286	96	85	
Age (year) ^a^				
Mean + SEM	49.3 ± 13.1	39.3 ± 7.8	60.6 ± 7.6	
Range	4–81	4–49	49–81	
Follow-up ^b^ (months)				
Mean ± SEM	44.7 ± 47.5	50.2 ± 53.3	34.5 ± 39.9	
Range	1.9–204	4–204	1.9–180	
Breast Cancer Subtype				
Breast Carcinoma ^c^	61.7%	36.5%	49.4%	0.192
DCIS	1.7%	1.0%	2.4%	
IDC	28.9%	52.1%	35.3%	
ILC	3.1%	4.2%	5.9%	
Metaplastic	2.8%	3.1%	5.9%	
Sarcoma	1.1%	1.0%	1.2%	
Other	0.7%	2.1%	0%	
Stage at Diagnosis				
Stage 0	1.9%	0%	3.7%	0.005
Stage I	13.0%	14.8%	11.1%	
Stage II	46.3%	29.6%	63.0%	
Stage III	31.5%	51.9%	11.1%	
Stage IV	7.4%	3.7%	11.1%	
Bilateral Breast Cancer	12.4%	16.1%	7.3%	0.229
Second Primary	27.3%	19.4%	37.5%	0.222
Developed Metastatic Disease	34.3%	38.5%	28.6%	0.445
Dead at last follow-up	46.7%	50%	41.7%	0.520

^a^ Age was not reported in 104 of cases; ^b^ Follow-up from breast cancer diagnosis; ^c^ Breast cancer subtype not specified

**Fig. 2:**
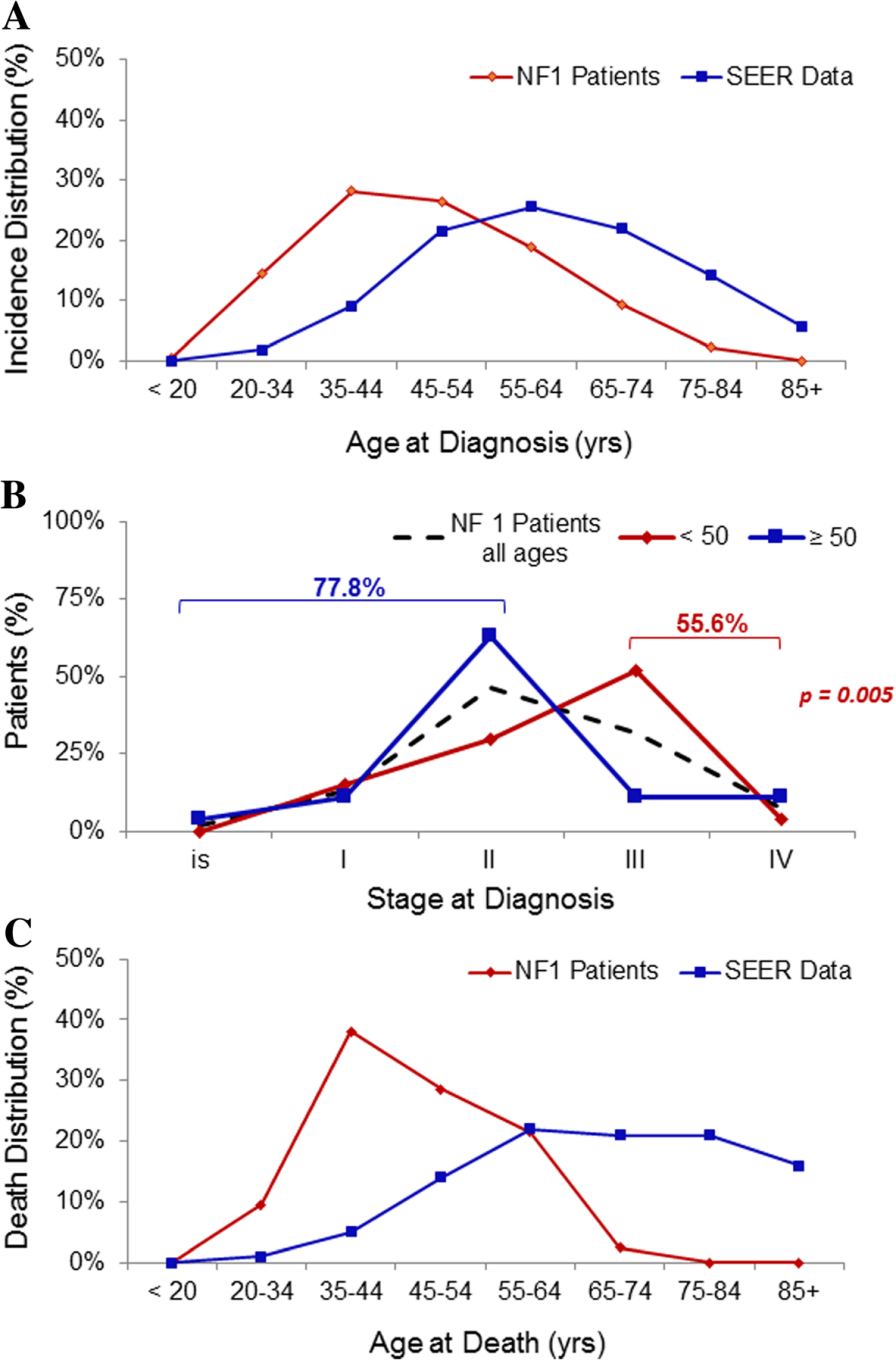
Descriptive analysis of breast cancer cases reported. **a** Age distribution of the incidence of breast cancer identified in 181 NF1 patients compared to the SEER database reported breast cancer age distribution of incident cases. **b** Distribution of breast cancer stage at time of diagnosis of breast cancer identified in 54 NF1 patients. **c** Age distribution of the incidence of breast cancer death identified in 76 NF1 patients compared to the SEER database reported breast cancer age distribution of deaths

Of the 286 cases identified, 181 reported the age of the patient. Mean age at breast cancer diagnosis was 49.3 years with a median age of 46 years and an interquartile range of 38.3 to 58.0 years. The majority of the patients were <  50 years of age with 53% <  50 years old, 28% between 35 and 44 years old and 15% < 35 years old. Age distribution of the incidence of breast cancer identified in these NF1 cases was evaluated based on their age at the time of diagnosis and revealed a peak age of diagnosis between the ages of 34 to 44 years. This incidence of breast cancer cases was compared to the SEER database for reported breast cancer age distribution of incident cases, which demonstrated a peak age of diagnosis between the ages of 55 to 64 years in the general population (Fig. 2a).

The type of breast cancer was reported in 110 cases; invasive ductal carcinoma (IDC) was the most commonly reported breast cancer subtype (75.5%). The other 176 breast cancer cases were identified as breast carcinoma. Breast cancer stage at time of diagnosis was reported in 54 cases (27 cases of patients < 50 years of age and 27 cases of patients ≥50 years of age). When analyzing all the female NF1 patients with breast cancer, the majority presented with stage I or II disease. However, when separated by age, women < 50 years of age presented with more advanced disease compared to those ≥50 years of age (56% vs. 22% stage III-IV, respectively, *p* = 0.005; Fig. 2b).

**Fig. 3:**
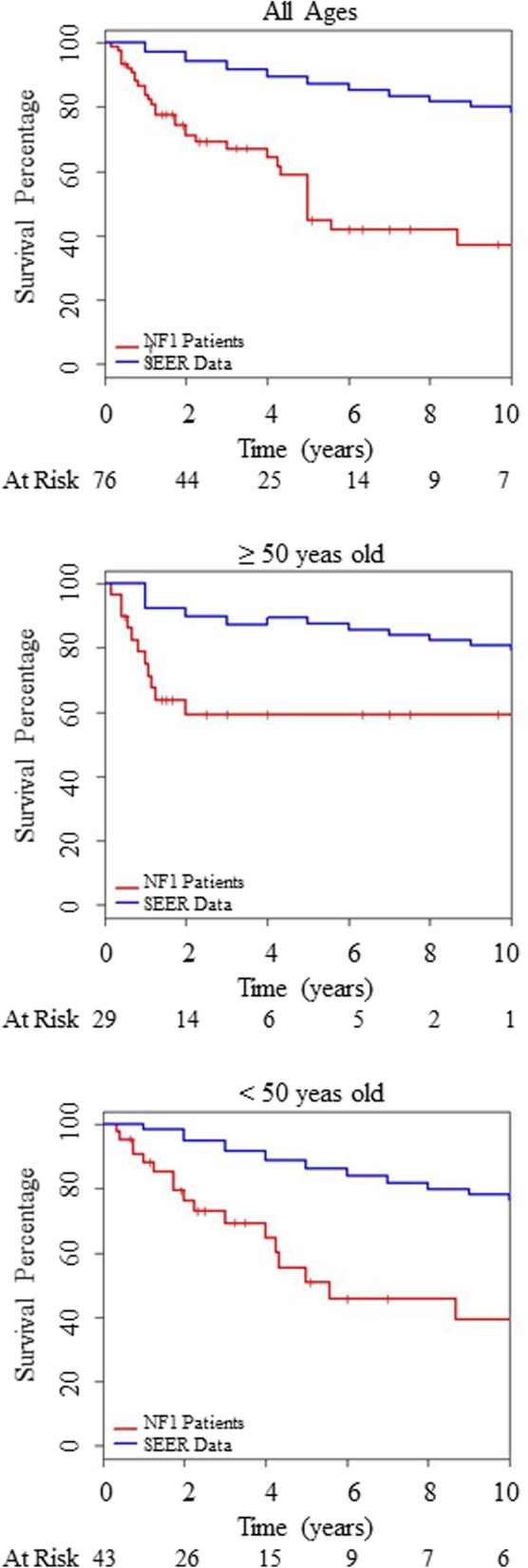
Relative breast cancer survival. Kaplan-Meier curves were used to estimate survival probabilities for the collected NF1 cases, and compared to the matched survival probabilities of general population from SEER database. Tick marks represent data censored at the last time the patient was known to be alive. Top panel represents 76 female NF1 patients of all ages. Middle panel represents 29 female NF1 patients aged 50 years or older. Bottom panel represents 43 female NF1 patients aged less than 50 years of age

Follow-up information was provided in 76 of the reported cases. Mean follow-up time was 44.7 months with a range of 1.9 to 204 months. Nearly half of the reported patients were dead at the time of last follow-up. Mean age at breast cancer death was 45.7 years with a median age of 48.5 years and an interquartile range of 41.3–59.7 years. The majority of the patients were <  50 years of age at time of breast cancer death with 64% < 50 years old, 38% between 35 and 44 years old and 10% < 35 years old. Age distribution of the incidence of breast cancer death identified in these NF1 breast cancer cases was evaluated based on their age at the time of death and revealed a peak age of death between the ages of 34 to 44 years. This incidence of breast cancer deaths was compared to the SEER database for reported breast cancer age distribution of deaths, demonstrating a peak age of death between the ages of 55 to 64 years in the general population (Fig. 2c). The relative survival of the female NF1 patients with breast cancer identified in this study was compared to the general population using the SEER database controlling for age and year at diagnosis for each patient (Fig. 3). The median survival for this entire NF1 breast cancer cohort was 5 years compared to the reported median breast cancer survival of over 20 years in the general population using the SEER database. When separated by age, the median survival of the female NF1 patients with breast cancer identified in this study was 5.58 years in those < 50 years of age and over 15 years in those ≥50 years of age.

### Meta-analysis characteristics of included studies

Among the four included studies, two were from the United States of America (USA) and two were from the United Kingdom (UK). These studies involved a total of 4178 female participants with NF1 who were being followed and documented 87 reported cases of breast cancer. The characteristics of these studies are summarized in Table 2. All included studies identified patients with NF1 via analyses of medical records, genetic registries, or national databases. The diagnoses of breast cancer were made via linked analyses of cancer registries, medical records, or national databases. All the included studies adjusted for age as a potential confounding factor. In quality assessment, the Newcastle-Ottawa Scale score of each included study ranged from 6 to 8 stars.

**Table 2:** Characteristics of studies included in meta-analysis

Author, Year	Sharif et al., 2007 [9]	Wang et al., 2012 [20]	Madanikia et al., 2012 [18]	Seminog et al. (2015) [21]
Country	United Kingdom	United States	United States	United Kingdom
Type of Study	Retrospective population-based cohort	Retrospective cohort	Retrospective cohort	Retrospective population-based case-controlled cohort
NOS Score				
Selection	⋆⋆⋆	⋆⋆⋆⋆	⋆⋆	⋆⋆
Comparability	⋆⋆	⋆	⋆⋆	⋆⋆
Outcome/Exposure	⋆⋆⋆	⋆⋆	⋆⋆	⋆⋆
Study Period	1975–2005	1990-2009	1995–2010	1999–2011
NF1 Patient Identification	Regional Genetic Service registry	Medical Genetics Clinic database and Henry Ford Hospital electronic medical records	Johns Hopkins Comprehensive Neurofibromatosis Center Registry	England NHS Information Center database and Center for National Statistics
Breast Cancer Diagnosis	North Western Cancer Registry	Metropolitan Detroit Cancer Surveillance System database	Johns Hopkins medical records and Oncology Clinical Information System	England NHS Information Center database and Center for National Statistics
Start of Follow-Up	January 1, 1975 or 20th birthday	January 1, 1973 or 20th birthday	20th birthday	Exposure: 1st hospital record for NF1Control: 1st hospital record of minor medical condition
End of Follow-up	Date of breast cancer diagnosis, last follow-up, or death	Date of breast cancer diagnosis or death	Date of breast cancer diagnosis or death	First hospital record for breast cancer or death
No. of Cases/Sample Size	14/304	11/76	4 / 126	58 / 3672
Age (years): Risk Estimates (95% CI)	SIR All ages: 3.5 (1.9–5.9) < 50: 4.9 (2.4–8.8)	SIR All ages: 5.2 (2.4–9.8) ≥ 50: 2.8 (0.6–8.2) < 50: 8.8 (3.2–19.2) 40–49: 4.4 (0.5–16.1) 30–39: 19.5 (5.3–50)	SIR All ages: 1.71 (0.54–4.12) ≥ 50: 0.81 (0.04–4.01) < 50: 2.68 (0.68–7.29)	RR 30–39: 6.5 (2.6–13.5) 40–49: 4.4 (2.5–7.0) 50–59: 2.6 (1.5–4.2) 60–69: 1.9 (1.0–3.3) 70–79: 0.8 (0.2–2.2)
Adjusted Factors	Age, calendar-period	Age	Age, calendar-period, race, time period of incidence	Age, calendar-period, region of residence, quintile of socioeconomic status

*NOS* Newcastle-Ottawa Scale, *SIR* standardized incidence ratio, *RR* relative risk, *CI* confidence interval

### Meta-analysis results

**Fig. 4:**
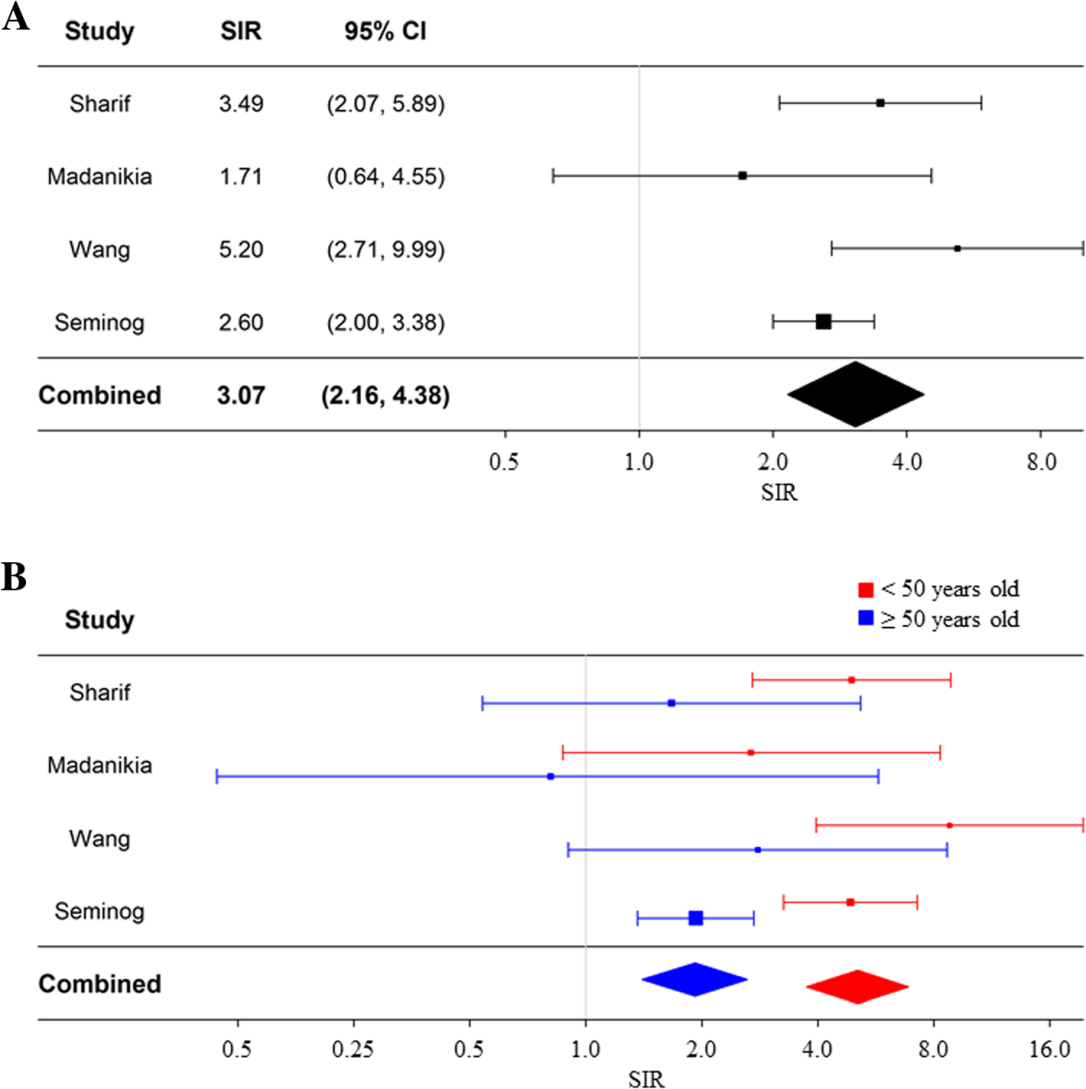
Standardized incidence ratio of breast cancer in women with NF1. Random effects meta-analysis models were used to generate Forest plots showing the relationship between NF1 and the risk of female breast cancer for all female patients with NF1 (**a**) and by age groups (**b**). Squares represent the risk estimate for each individual study. Horizontal lines represent 95% confidence interval. Diamonds represent the summary risk estimate with 95% confidence interval. SIR, standardized incidence ratio. CI, confidence interval

The unadjusted SIRs for each study and the combined SIR of breast cancer in women with NF1 are presented in Fig. 4. Based on a random-effects model, the combined SIR of breast cancer for all women with NF1 was 3.07 (95% CI: 2.16–4.38). We observed moderate heterogeneity between studies for all women (I^2^ = 44%). However, women with NF1 < 50 years of age demonstrated a higher SIR of 5.08 (95% CI: 3.77–6.81) compared to 1.92 (95% CI: 1.40–2.63) in those ≥50 years of age. For both age groups, very low heterogeneity was observed with I^2^ values less than 5%. Similar results were found using a fixed-effects model (data not shown).

## Discussion

The association between NF1 and increased risk of malignant tumors has been widely described with the most commonly reported associations being gliomas and malignant peripheral nerve sheath tumors [10–14]. Case reports and several cohort and epidemiological studies have described an association of breast cancer with NF1. The first case of a female patient with NF1 and a breast malignancy identified in this study was reported in 1933 by Arthur Jackson, where a 52 year old female with a neurogenic sarcoma of the left breast was presented [35]. Since then, 41 additional case reports/case series have been reported describing an additional 74 females with NF1 that developed breast cancer [17, 36, 38–41, 44–50, 53–67, 71–75, 77–84]. A total of 16 cohort studies evaluating breast cancer in NF1 patients were identified with a total of 211 cases of female breast cancer reported [9, 10, 18–22, 37, 42, 43, 51, 52, 68–70, 76].

Descriptive analysis of these 286 reported cases of NF1 and female breast cancer demonstrated a median age of 46 years at time of breast cancer diagnosis, compared to 62 years in the general population reported in the SEER database. The peak age of breast cancer diagnosis in these NF1 women was between 34 to 44 years. NF1 women younger than 50 years of age were found to have more advanced disease at the time of their breast cancer diagnosis (56% presenting with stage III-IV disease) compared to those 50 years of age or older (22% presenting with stage III-IV disease). The median survival for all of the identified cases of NF1 women with breast cancer was 5 years compared to the reported median breast cancer survival of over 20 years in the general population using the SEER data base. The median age at time of breast cancer death was found to be 48.5 years in this group of NF1 breast cancer cases compared to 68 years in the general population reported in the SEER database. These findings suggest that there is a high incidence of breast cancer in NF1 women younger than 50 years of age and that these women tend to present with more advanced disease and possibly experienced an increased breast cancer related mortality. However, it is important to note that true comparison with SEER database is difficult with this cohort of patients as these patients originated from 23 different countries and the survival reported in the USA SEER database may be better than in some other countries included in this cohort.

To our knowledge, this study is the first meta-analysis to evaluate the association between women with NF1 and the risk of developing breast cancer. Findings of this meta-analysis of 4 cohort studies, following a total of 4178 female patients with NF1, indicate that women with NF1 have a three-fold increased risk of developing breast cancer compared to the general population (SIR = 3.07; 95%CI 2.16–4.38). Additionally, a higher-than-expected number of breast cancer cases were identified in women with NF1 who were younger than 50 years of age, giving a five-fold increased risk of breast cancer in this age group compared to women younger than 50 years old in the general population (SIR = 5.08; 95%CI 3.77–6.81). Women with NF1 who were 50 years of age and older demonstrated a smaller increased risk of breast cancer compared to women 50 years and older in the general population. This lower increased risk of breast cancer in older NF1 patients may be due to the fact that women with NF1 develop breast cancer at a younger age than the general population. Given this early elevated risk a fair number of the susceptible NF1 population may develop breast cancer prior to 50 years of age, thereby decreasing the number of susceptible women in the older population.

In addition to the four studies included in this meta-analysis, several other cohort and epidemiological studies have suggested an association of breast cancer in young women with NF1. In 1972 Brasfield et al. described a cohort of 110 NF1 patients from the USA looking at the biologic behavior and the natural history of this disease. Herein, he found that 5 of 54 (9.3%) females, one of them 39 years old, developed breast cancer and noted that all patients with breast cancer died within 5 years of the breast cancer diagnosis [22]. Following this study, Sorensen et al. described a 42 year follow-up on a nation-wide cohort of 212 NF1 patients from Denmark and found that 7 of 88 (8.0%) females developed breast cancer [42]. In 1988 Huson et al. published a population-based study in south-east Wales to determine the prevalence of NF1 and its complications and identified that 1 of 75 (1.3%) females developed breast cancer; that patient was only 43 years old [43]. Zoller et al. conducted a long term follow-up study of 70 adult NF1 patients previously identified in a population-based study that were living in Goteborg, Sweden where 2 of 33 (6.1%) females were found to have developed breast cancer; of which, one was 38 years of age at the time of her diagnosis [19]. Nakamura et al. evaluated 26 female Japanese patients with NF1 and breast cancer and found an 18.5% incidence of breast cancer in NF1 women younger than 35 years old compared to a previously reported 6.7% incidence in the general Japanese female population among 1438 cases [49]. Kim et al. conducted a retrospective review of 125 NF1 patients to investigate the incidence and spectrum of malignant tumors in Korean NF1 patients and found that 3 of 62 (4.8%) female patients, one less than 50 years of age, developed breast cancer [70].

Walker et al. conducted a prospective population based cohort study of 448 NF1 patients (227 females) to evaluate the incidence and type of malignant tumors in this population in the UK. In this study, five (1.8%) females developed breast cancer and the risk of breast cancer was found to be significantly higher in NF1 patients younger than 50 years of age compared to this age group in the general population; SIR = 4.0 (95% CI: 1.1–10.3) [10]. This increased risk of breast cancer was not seen for NF1 patients 50 years of age or older; SIR = 0.59 (95% CI: 0.02–3.33) [10]. Of note, follow-up in this study was concluded at the time of diagnosis of the earliest first malignant cancer (not at the time of breast cancer diagnosis) and therefore was not included in the meta-analysis of this manuscript. A recently published study not included in the meta-analysis of this manuscript was conducted by Uusitalo et al. to evaluate the cancer incidence and mortality in a population-based cohort of 1404 (737 women) Finnish patients with NF1 [11]. In this study, thirty-one (4.2%) women developed breast cancer and the risk of breast cancer was found to be significantly higher in NF1 patients younger than 40 years of age compared to this age group in the general population; SIR = 11.1 (95% CI: 5.6–19.5) [11]. Additionally, Uusitalo et al. demonstrated that women with NF1 have a five-fold increased risk of breast cancer mortality compared to the general population with a SMR of 5.2 (95% CI: 2.4–9.9) and found that when breast cancer survival was analyzed alone, 5-year survival was poorer in patients with NF1 compared with those without NF1 (67.9% vs. 87.8%, respectively) [11].

Given the rarity of breast cancer events described, especially when divided by age groups, it is important to evaluate a large defined population. This meta-analysis was able to assess the risk of breast cancer in a large defined patient population. The strengths of the present meta-analysis include less influence exerted by small-study bias, a moderate-to-high quality of studies included in the meta-analysis, and very low heterogeneity observed for both age groups. However, this study has several limitations. First, the associations presented are unadjusted. Due to the lack of reporting across studies, other known confounding risk factors for breast cancer, such as medications, smoking, use of hormone replacement therapy, parity or other reproductive factors cannot be accounted for. Thus, one cannot exclude residual or unmeasured confounding as the alternative explanation of these results. Second, given that the number of available cohort studies was limited, this study has a potential to be limited by publication bias. It is possible that a number of neuro-fibromatosis centers have assessed breast cancer in their cohorts without finding evidence of an increased risk and that these findings have not been published. Third, referral bias may be present in two of the included studies [18, 20]. Referral bias of single institution studies of large tertiary centers may result from selective referral of malignant cases to these institutions. Thus, data from these single institution studies may not be representative of the whole population.

This systematic literature review and meta-analysis suggests that women with NF1 less than 50 years of age have a five-fold increased risk of breast cancer, present with more advanced disease, and may have an increased breast cancer related mortality. The findings of this study support the notion that it may be reasonable to consider NF1 in conjunction with other hereditary breast cancer syndromes. Early breast cancer screening guidelines need to be extended to include women with NF1. Given that this study demonstrated a peak age of breast cancer diagnosis between 35 and 44 years of age in these patients with NF1, this study provides further evidence for early breast cancer screening starting at 30 years of age in women with NF1. Guidelines recently produced by the National Comprehensive Cancer Network (NCCN) now suggested early screening for NF1 patients beginning at age 30 [85]. However, screening methods need to be carefully evaluated and weighed against the risk of radiation exposure to young women with NF1. Additionally, core biopsies of image-detected lesions may give a diagnosis of benign neurofibromas rather than breast cancer. The risk of over-diagnosis may be exacerbated by the lower specificity of MRI, although experienced breast radiologists should be able to distinguish neurofibroma from breast cancer in most instances [86].

With the recent improvement and reduced cost of DNA sequencing technology, the use of multigene panels for clinical genetic testing of patients with a high risk of hereditary breast cancer has increased [87]. The *NF1* gene is included in some of the breast cancer screening genetic panels as deleterious mutations on the *NF1* gene have been associated with a two to four fold increased risk of breast cancer [88]. The goal of these genetic panels are to help stratify of patients according to levels of risk, aid in family cancer genetic counseling, and provide guidance on the identification of women to whom early breast cancer screening, risk-reducing medication, and/or risk-reducing surgery should be offered [88]. However, with the use of multigene panels unexpected pathogenic variants or variants of uncertain significance can be identified for which clinical significance of increased cancer risk is unknown. Multigene panel breast cancer genetic testing may identify mutations in the *NF1* gene in patients not previously known to have NF1. Given that approximately 50% patients with NF1 have de novo mutations [89], patients who are identified in this manner should be carefully assessed for subtle features with NF1.

The *NF1* gene has been implicated as a breast cancer driver with somatic mutations reported in 27.7% of all breast carcinomas [15, 16]. Previous studies have suggested that a mutation in the *NF1* gene may also result in, or predispose cells to, a mutation in other genes on that same chromosome [90]. The *NF1* gene and *BRCA1* gene are both on chromosome 17, about 20 centiMorgan (cM) apart, and it has been suggested that there may be an interaction between these two genes [58, 81]. However, the risk of breast cancer in patients found to have a variant in the *NF1* gene without any clinical evidence of NF1 is not clear.

Couch et al. recently evaluated the risk of breast cancer associated with inactivating variants of the *NF1* gene, along with several other genes associated with increased risk of breast cancer, identified by clinical genetic testing of patients with breast cancer. After exclusion of *BRCA1*, *BRCA2*, and syndromic breast cancer genes (*CDH1*, *PTEN*, and *TP53*), observed pathogenic variants in *ATM*, *BARD1*, *CHEK2*, *PALB2* and *RAD51D* were associated with high or moderately increased risks of breast cancer [91]. But, pathogenic variants in the NF1 gene were not associated with increased risks of breast cancer [91]. Several other studies assessing the risk of breast cancer with multi-gene panels have also failed to demonstrate an association with *NF1* pathogenic variants and an increased risks of breast cancer [92–94]. However, Evans et al. warn about the potential pitfalls of using commercial multi-gene panels to confirm syndromic associations with cancers, in particularly NF1 and breast cancer [95]. Their review discussed two main reasons why pathogenic variants in the *NF1* gene may have not been associated with an increased risks of breast cancer; 1) it is likely that patients with NF1 are selected out of testing due to their know diagnosis or other socioeconomic factors and 2) lack of appropriate controls [95]. Additionally, a study conducted by Frayling et al. evaluating *NF1* constitutional mutation types and breast cancer risk in patients with NF1 and breast cancer showed that different *NF1* variants demonstrated different risks of breast cancer and that nonsense and missense mutations may be associated with a higher breast cancer risk [96].

## Conclusions

The increased risk of breast cancer found in this meta-analysis and other previous studies reinforces that increased attention to the breast cancer risk in young women with NF1 is needed. Appropriate physician and patient education with increased awareness of the association of early onset breast cancer in patients with NF1 and the need for early breast cancer screening is crucial for this patient population. Continued education of the general public and primary health care providers will allow for appropriate implementation of the most recent NCCN breast cancer screening guidelines in all young women with NF1 starting at 30 years of age. Furthermore, additional studies are required to assess the influence of *NF1* pathogenic variants identified in patients undergoing clinical genetic testing on breast cancer risk in individuals without clinical evidence of NF1.

## Additional file

Additional file 1: Meta-analysis of Observational Studies in Epidemiology (MOOSE) Checklist. Checklist with brief description of how research criteria of observational studies in epidemiology were handled for reporting of background, search strategy, methods, results, discussion, and conclusion in the meta-analysis. (PDF 306 kb)

## Abbreviations

CI: Confidence interval
HR: Hazard ratio
MOOSE: Meta-analysis of Observational Studies in Epidemiology
NCCN: National Comprehensive Cancer Network
NF1: Neurofibromatosis type I
NOS: Newcastle-Ottawa Scale
OR: Odds ratios
RR: Relative risk
SEER: Surveillance Epidemiology and End Results
SIR: Standardized incidence ratio
