# Supplementary material for: Increased breast cancer risk in women with neurofibromatosis type 1: a meta-analysis and systematic review of the literature
Source: Hered Cancer Clin Pract. 2019 Mar 25;17:12. doi: 10.1186/s13053-019-0110-z (PMC6434896; doi:10.1186/s13053-019-0110-z)
Supplement: Supplementary file 1 — Meta-analysis of Observational Studies in Epidemiology (MOOSE) Checklist. Checklist with brief description of how research criteria of observational studies in epidemiology were handled for reporting of background, search strategy, methods, results, discussion, and conclusion in the meta-analysis. (PDF 306 kb) [file 13053_2019_110_MOESM1_ESM.pdf]

Additional File 1. Meta-analysis of Observational Studies in Epidemiology (MOOSE) Checklist

	Criteria	Brief description of how the criteria were handled in the meta-analysis
**Reporting of background should include**		
√	Problem definition	A link between Neurofibromatosis type 1 (NF1) and breast cancer has been suggested in several cohort and epidemiological studies and numerous cases of patients with NF1 presenting with breast cancer have been reported. The strength of the association between NF1 and the increased breast cancer risk remains uncertain due to the small study populations and differences in participants and methodological methods used in the previous studies.
√	Hypothesis statement	Women with NF1 have a higher risk of breast cancer compared to the general population.
√	Description of study outcomes	Development of breast cancer
√	Type of exposure or intervention used	Diagnosis of NF1
√	Type of study designs used	Observational study
√	Study population	Women with NF1 and controls
**Reporting of search strategy should include**		
√	Qualifications of searchers (eg, librarians and investigators)	The credentials of the investigators are provided in the author list.
√	Search strategy, including time period included in the synthesis and keywords	PubMed and PMC from oldest record to December 2015.<br><br>Key words: "Neurofibromatoses", "Neurofibromatosis 1", "genes, Neurofibromatosis 1", and "Neurofibromatosis type 1" in combination with "breast neoplasms", “breast cancer”, “malignancy”, “neoplasm”, “tumor”, or “cancer.”<br><br>The search was restricted to studies in human beings and publications in English language.
√	Effort to include all available studies, including contact with authors	The references of all retrieved articles and recent reviews were also manually reviewed.
√	Databases and registries searched	PubMed and PMC
√	Search software used, name and version, including special features used (eg, explosion)	We did not employ the use of special search software.
√	Use of hand searching (eg, reference lists of obtained articles)	References of all retrieved articles and recent reviews were reviewed.

√	List of citations located and those excluded, including justification	Details of the literature search process are outlined in the flow chart.
√	Method of addressing articles published in languages other than English	The search was restricted to publications in English language.
√	Method of handling abstracts and unpublished studies	The search was restricted to published studies and abstracts without full text and unpolished studies were excluded.
	Description of any contact with authors.	No attempt was made to contact any authors.
**Reporting of methods should include**		
√	Description of relevance or appropriateness of studies assembled for assessing the hypothesis to be tested	The inclusion criteria are presented in the "Study Selection" section.
√	Rationale for the selection and coding of data (eg, sound clinical principles or convenience)	The following data from each included study were extracted: first author, publication year, country, study design, sample size, number of cases / controls, diagnostic criteria, age at time of breast cancer diagnosis, follow-up duration, breast cancer stage at time of diagnosis, breast cancer subtype, development of bilateral breast cancer, development of other primary cancers, development of metastatic breast cancer, survival outcome, and effect sizes (SIR, RR, OR, HR) with 95% CI and adjusted factors.
√	Documentation of how data were classified and coded (eg, multiple raters, blinding, and inrerrater reliability)	Data were independently extracted and analyzed by two investigators and final decision was reached by consensus.
√	Assessment of confounding (eg, comparability of cases and controls in studies where appropriate)	Table 2 presents the characteristics of studies included in the meta-analysis and adjustment factors for each study.
√	Assessment of study quality, including blinding of quality assessors; stratification or regression on possible predictiors of study results	The quality of each study was assessed using the Newcastle-Ottawa Scale.
√	Assessment of heterogeneity	I^2 statistic were used to explore the heterogeneity among studies.
√	Description of statistical methods (eg, complete description of fixed or random effects models,	Descriptions of statistical methods used are detailed in the "Statistical analysis" section.

	justification of whether the chosen models account for predictors of study results, dose-response models, or cumulative meta-analysis) in sufficient detail to be replicated	
√	Provision of appropriate tables and graphics	Two main tables and one supplemental table are provided. One flow chart and two forest plots appear in the main text.
**Reporting of results should include**		
√	Graph summarizing individual study estimates and overall estimate	Figure 4
√	Table giving descriptive information for each study included	Table 2
	Results of sensitivity testing (eg, subgroup analysis)	We performed a subgroup analysis based on two age groups (< 50 or ≥ 50 years of age). Given that a small number of studies were included in the meta-analysis, we were selective about what subgroups/other factors to examine. We did not have any drastically different studies that we might have wanted to exclude and check sensitivity of the estimates.
√	Indication of statistical uncertainty of findings	95% confidence intervals are presented with all summary effect estimates.
**Reporting of discussion should include**		
	Quantitative assessment of bias (eg, publication bias)	Quantitative assessment of publication bias was not performed. Publication bias here would arise from either of these cohorts if they are published because of high rates of cancer or other cohorts are not published because of low/normal rates. Since we aren't evaluating a treatment effect, the suppression of null studies is less of a concern here. We also limited ourselves to these 4 studies to avoid potential publication bias from case studies/series. We could make a funnel plot; however, with only 4 studies, it would not be any more informative than the forest plot that we already present.
√	Justification for exclusion (eg, exclusion of non-English-language citations)	The details of the exclusion of studies are shown in Flow chart.
√	Assessment of quality of included studies	Tables 2.
**Reporting of conclusions should include**		
√	Consideration of alternative explanations for observed	We discussed that we cannot exclude chance, residual or unmeasured confounding as alternative explanation for our

	results	findings.
√	Generalization of the conclusions (ie, appropriate for the data presented and within the domain of the literature review)	We discussed that the results of current study suggests that women with NF1 less than 50 years of age have a fivefold increased risk of breast cancer, present with more advanced disease, and may have an increased breast cancer related mortality.
√	Guidelines for future research	We discussed that a large multi-center, long-term, follow-up prospective study or a national initiative should be conducted to better delineate the true risk of breast cancer in NF1, understand the etiology and natural history of breast cancer in this population, and to determine the optimal screening method and timing to allow for earlier breast cancer diagnosis and decreased breast cancer associated morbidity and mortality in women with NF1.
√	Disclosure of funding source	The funding information is shown in the text.
